# Intensification of CO_2_ absorption using MDEA-based nanofluid in a hollow fibre membrane contactor

**DOI:** 10.1038/s41598-021-82304-2

**Published:** 2021-01-29

**Authors:** Yan Cao, Zia Ur Rehman, Nayef Ghasem, Mohamed Al-Marzouqi, Nadia Abdullatif, Ali Taghvaie Nakhjiri, Mahdi Ghadiri, Mashallah Rezakazemi, Azam Marjani, Mahboubeh Pishnamazi, Saeed Shirazian

**Affiliations:** 1 School of Mechatronic Engineering, Xi’an Technological University, Xi’an, 710021 China; 2 Department of Chemical & Petroleum Engineering, UAE University, AL-Ain, UAE; 3 Department of Petroleum and Chemical Engineering, Science and Research Branch, Islamic Azad University, Tehran, Iran; 4 Institute of Research and Development, Duy Tan University, Da Nang, 550000 Vietnam; 5 The Faculty of Environment and Chemical Engineering, Duy Tan University, Da Nang, 550000 Vietnam; 6 Faculty of Chemical and Materials Engineering, Shahrood University of Technology, Shahrood, Iran; 7 Department for Management of Science and Technology Development, Ton Duc Thang University, Ho Chi Minh City, Vietnam; 8 Faculty of Applied Sciences, Ton Duc Thang University, Ho Chi Minh City, Vietnam; 9 The Faculty of Pharmacy, Duy Tan University, Da Nang, 550000 Vietnam; 10 The Faculty of Environmental and Chemical Engineering, Duy Tan University, Da Nang, 550000 Vietnam; 11 Laboratory of Computational Modeling of Drugs, South Ural State University, 76 Lenin prospekt, 454080 Chelyabinsk, Russia

**Keywords:** Engineering, Chemical engineering, Environmental chemistry, Theoretical chemistry

## Abstract

Porous hollow fibres made of polyvinylidene fluoride were employed as membrane contactor for carbon dioxide (CO_2_) absorption in a gas–liquid mode with methyldiethanolamine (MDEA) based nanofluid absorbent. Both theoretical and experimental works were carried out in which a mechanistic model was developed that considers the mass transfer of components in all subdomains of the contactor module. Also, the model considers convectional mass transfer in shell and tube subdomains with the chemical reaction as well as Grazing and Brownian motion of nanoparticles effects. The predicted outputs of the developed model and simulations showed that the dispersion of CNT nanoparticles to MDEA-based solvent improves CO_2_ capture percentage compared to the pure solvent. In addition, the efficiency of CO_2_ capture for MDEA-based nanofluid was increased with rising MDEA content, liquid flow rate and membrane porosity. On the other hand, the enhancement of gas velocity and the membrane tortuosity led to reduced CO_2_ capture efficiency in the module. Moreover, it was revealed that the CNT nanoparticles effect on CO_2_ removal is higher in the presence of lower MDEA concentration (5%) in the solvent. The model was validated by comparing with the experimental data, and great agreement was obtained.

## Introduction

Fossil fuel consumption has been increased over the last 150 years to meet its demand for power generation. However, rising fuel consumption has caused considerable emissions of greenhouse gases especially carbon dioxide (CO_2_), which is one of the current major issues in the world dealing with environmental impact. It can lead to climate change, and a gradual increase in globe temperature and consequently has a negative impact on the world population, environmental health and economic development^1^. Therefore, development of effective and environmental-friendly technology to store or capture CO_2_ has great significance from the environmental point of view. Various CO_2_ capture procedures, including physical and chemical absorption^2^, solid adsorption^3^, cryogenic^4^ and membrane technology^5^ have been utilized to treat gas streams containing CO_2_ and other pollutant gases.

Chemical absorption in aqueous amine solvents is the most common approach for CO_2_ capture at industrial scale because of its advantages such as high efficiency and mature process^6^. Methyldiethanolamine (MDEA) is a tertiary amine and cost-effective absorbent which is widely used for CO_2_ removal owing to its acceptable CO_2_ absorption capacity, carbamate formation, low corrosion rate, and lower heat regeneration^7^. There is direct contact between amine solutions and gas phase in conventional systems, causing some difficulties, including weeping, foaming, entrainment, flooding, and excessive loading. Furthermore, high energy consumption and their complexity are their main drawbacks. Membrane technology can be utilized as a promising method for the separation of CO_2_ and other gaseous pollutants from various gas mixtures to overcome the drawbacks of traditional separation techniques^8^.

Dispersion of nanoparticles (NPs) into either organic or aqueous solvents has been proposed as one of the useful ways to enhance CO_2_ absorption^7,9^. It has been revealed that CO_2_ removal enhances by 4.5% and 5.6% when there is Al_2_O_3_ and SiO_2_ NPs in methanol solvent at 20 °C, respectively^9^. The mass transfer coefficient for CO_2_ absorption would be improved when NPs are added to the absorbent solution. The interphase boundary layer mixing because of the presence of NP and Grazing effect phenomena occurs when NPs are properly dispersed in the solvent media^10–13^. NPs dispersion increases the fluid turbulence in the gas–liquid boundary layer and subsequently improves CO_2_ mass transfer rate^14^. Grazing effect means a strong affinity of CO_2_ molecules into dispersed NPs and then reduction of gas concentration in the solvent, which can increase driving force between liquid and gas phases^15^. Brownian motion of NPs in the solvent results in inducing micro-convections in the liquid which would improve the mass transfer performance of the employed process^16^.

In terms of theoretical evaluation, mechanistic transport phenomena modeling of hollow fibre membrane contactor have been widely investigated to understand the effect of operating conditions, membrane specification, solvent type, and etc.^17–19^. Indeed, model-based process development approach has been employed for process intensification and improvement of separation efficiency. However, in the previous developed models, Grazing and Brownian motion of NPs effects which happen in the presence of NPs were not considered in detail.

A DEAB-based nanofluid (NF) for carbon dioxide removal was studied in a hollow fibre membrane contactor, confirming the dispersion of carbon nanotube (CNT) and SiO_2_ NPs considerably increase the removal rate. The effect of adding a number of NPs to water on the CO_2_ absorption was investigated by Peyravi et al.^20^ and it was found that solvent velocity and NP content had significant influences on CO_2_ removal from the gas phase. As it can be seen, using NPs in solvent for improvement of CO_2_ absorption in Hollow-Fiber Membrane Contactors (HFMCs) is increasing. Therefore, it will be highly important to investigate these systems to obtain a detailed understanding of the process. Computational fluid dynamics (CFD) is recognized as a sophisticated computational technique for understanding transport phenomena in different processes, and has attracted much attention.

In the current work, a mechanistic model and simulation were developed to investigate NPs presence in an amine solvent (MDEA) on the CO_2_ absorption in a HFMC. Changes in CNT concentration and operation conditions on the CO_2_ mass transfer rate were studied in terms of carbon dioxide recovery from the gas stream. A numerical method based on finite element discretization scheme was implemented to solve the nonlinear system’s equations. The modeling outputs were compared with measured results to evaluate validity of the developed mechanistic method.

## Experimental

**Figure 1:**
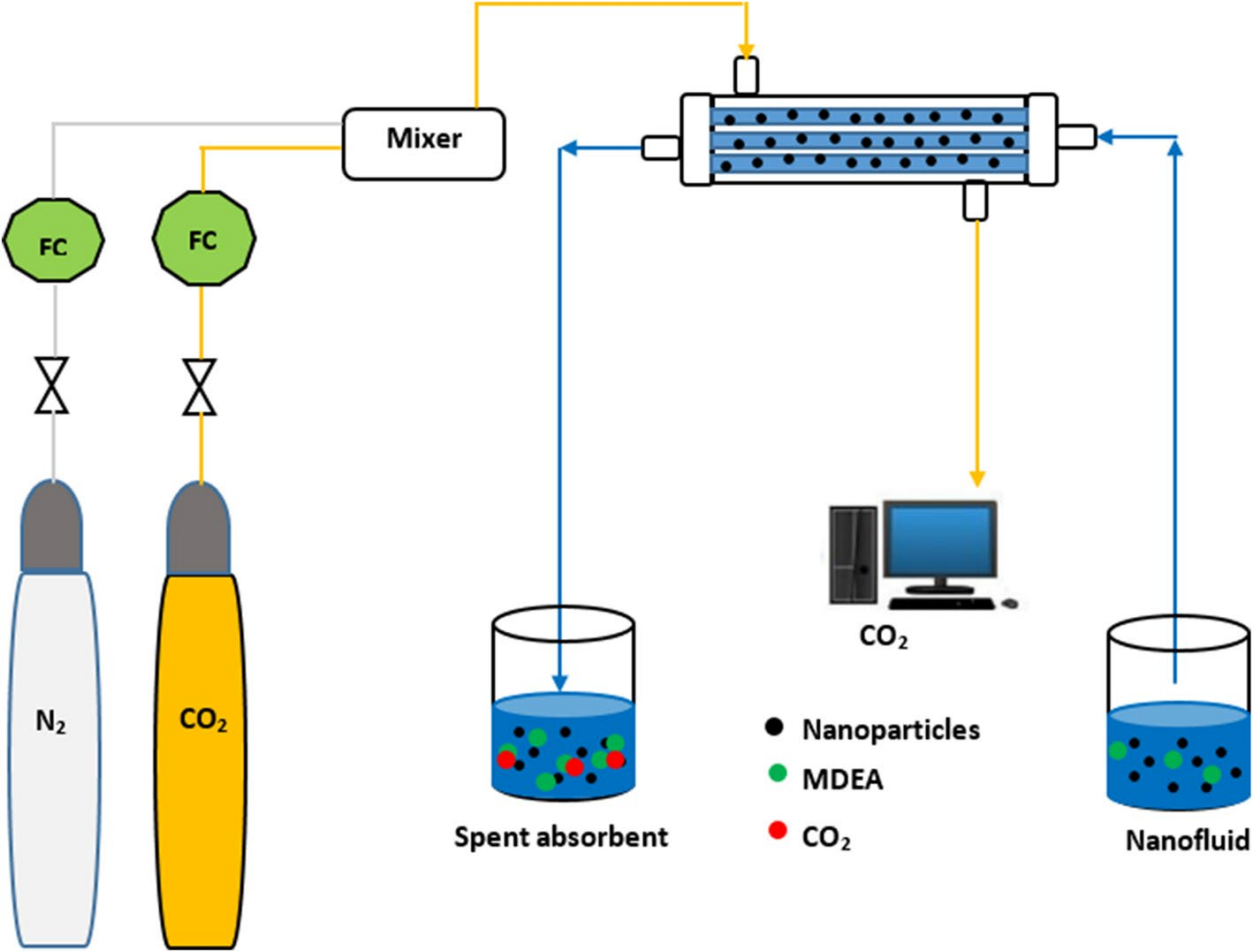
Schematics of the CO_2_ absorption setup using nanofluid.

The polyvinylidene fluoride (PVDF) fibres were made using the thermally induced phase inversion technique. Then, the prepared PVDF fibres were packed in perspex glass as a shell of the module. It was supplied by Sign Trade. It should be pointed out that the blockage of the fibres was checked before packing into the module. Then, the top and bottom sides of the contactor were sealed by resin. The CNTs nanoparticles were provided by Cheap Tubes Ins. Company (USA). In CO_2_ absorption experiments, the gas phase flow rate was adjusted using a flow controller, whereas the liquid flow rate was set via a Masterflex L/S Digital Pump (Cole Parmer). At the exit of gas stream, a CO_2_ Analyzer (CAI-600 Seri, Gas Analyzers, USA) was employed to find the CO_2_ content in the gas stream. The gas-phase passed through the shell-side of HFMC, and the MDEA based NF flew through the tube section in a counter-current configuration. The volumetric flow rate of the CO_2_ and N_2_ mixture was measured using mass flow controller. The NPs were stable in the solution without adding any surfactant. The diagram of the used experimental system for membrane CO_2_ absorption was provided in Fig. 1. Experimental conditions are given in Table 1.

**Table 1:** Experimental conditions for membrane CO_2_ capture.

Parameter	Value
Temperature (K)	298
Base fluid	MDEA
Nanoparticle	CNT
Inlet CO_2_ concentration (vol%)	20
Nanoparticle concentration (wt%)	0.05–0.20
Gas phase flow rate (ml/min)	10–400
Solvent flow rate (ml/min)	10–40

To prepare the solvent for the gas absorption experiments, solution of MDEA was initially prepared at 3 different percentages of 5, 10, and 20 wt%. Then, 0.5 wt% of CNT particles was added to each sample and sonicated by the high-intensity ultrasonic liquid processor for 60 min to stabilize it. The mixture was then ready to be utilized in the experiments as solvent where no surfactant was required to stabilize the suspension^21–23^ . Table 2 lists properites of the PVDF hollow fiber membrane used in the absorption process in this study.

**Table 2:** Characteristics of PVDF hollow fiber member module used in this work.

Parameters	value
Fiber length **(**mm**)**	210
Number of membranes	11
Inner diameter (mm)	0.42
Outer diameter (mm)	1.1
Membrane thickness (mm)	0.34
Membrane porosity	0.4585
Module contact area (m^2^)	0.003
Module inner diameter (cm)	0.8
Module outer diameter (cm)	1.2

## Model development

**Figure 2:**
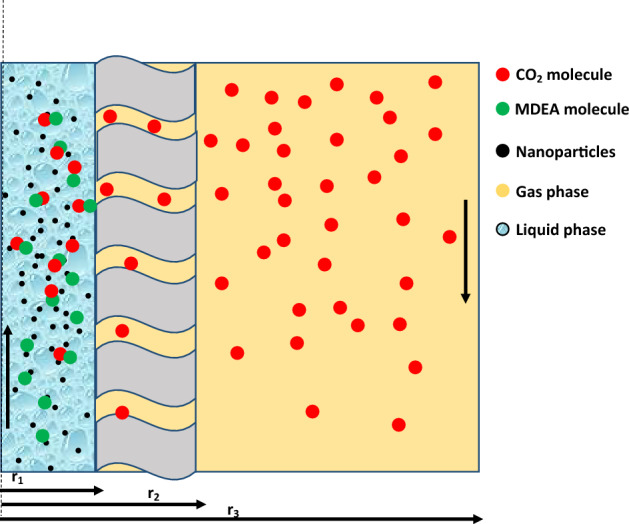
A schematic diagram of a single hollow fibre for CO_2_ removal.

Figure 2 illustrates a schematic demonstration of CO_2_ absorption process into MDEA-based NF using a microporous/hydrophobic (non-wetted) PVDF contactor module. To simplify the model development, one fibre is considered, which is then divided into three subdomains: i.e. tube subdomain (NF), polymeric membrane, and shell subdomain (N_2_ and CO_2_ mixture). As can be observed from Fig. 2, MDEA-based NF flows in the tube subdomain, whereas the gas mixture fed to the shell subdomain in the opposite direction. The mechanistic model is built axis-symmetrically (two dimensional) because of the non-existence of angular gradient^6^. Here, we used Happel’s formula to estimate the shell side radius (Fig. 2) around the fibre^24^:

1 $$r_{3} = \left( {\frac{1}{1 - \emptyset }} \right)^{0.5} r_{2}$$

2 $$1 - \emptyset = \frac{{nr_{2} }}{{{\mathcal{R}}^{2} }}$$

where φ denotes the void fraction. The membrane specification was provided in Table 3. The following assumptions are used for process modeling:

- Steady-state and isothermal fluid flow.
- Gas velocity profile is fully developed.
- Ideal gas behavior was considered.
- Laminar flow for liquid phase in the system.
- Gas mixture only fills the fiber pores at all operating conditions.

**Table 3:** Membrane, module, fluid and NPs specifications^25^.

Parameter	Unit	Value
Material	PVDF	–
Membrane inner diameter (${d}_{1}$)	m	4.2 × 10^–4^
Membrane outer diameter (${d}_{2}$)	m	11 × 10^–4^
Module inner diameter (${d}_{3}$)	m	0.008
Membrane thickness ($\updelta$)	m	3.4 × 10^–4^
Porosity ($\varepsilon$)	–	0.4585
Tortuosity (η)	–	5.14
${\text{D}}_{{{\text{CO}}_{{2}} ,{\text{shell}}}}$	m^2^/s	1.39 × 10^–5^
${\text{D}}_{{{\text{CO}}_{{2}} ,{\text{membrane}}}}$	m^2^/s	${\text{D}}_{{{\text{CO}}_{{2}} ,{\text{shell}}}}$(ε/η)
${\text{D}}_{{{\text{CO}}_{{2}} ,{\text{tube}}}}$	m^2^/s	1.45 × 10^–9^
Henry's law constant (*m*)	–	0.891
Nanoparticle true density	kg/m^3^	2200
Nanoparticle average size	nm	8
Nanoparticle morphology	–	Tubular

### Shell side's equations

The mathematical formula that describes the transport of CO_2_ molecules from the gas phase (N_2_ and CO_2_ mixture) to MDEA-based NF is the continuity equation, which can be expressed as^25,26^:

3 $$\frac{{\partial C_{{CO_{2} }} }}{\partial t} = - \nabla N_{{CO_{2} }} + R_{{CO_{2} }}$$

where *C*, *R* and *N* refer to the CO_2_ concentration, reaction rate, and mass flux, respectively. The mass transfer flux is calculated using^6,26^:

4 $$N_{{CO_{2} }} = - D_{{CO_{2} }} \nabla C_{{CO_{2} }} + C_{{CO_{2} }} V_{Z}$$

where *D* is the diffusivity and *V*_*z*_ is the velocity. The steady-state mass transfer equation in the shell side is then derived as^27^:

5 $$D_{{CO_{2} , s}} \left[ {\frac{{\partial^{2} C_{{CO_{2} ,s}} }}{{\partial r^{2} }} + \frac{1}{r}\frac{{\partial C_{{,CO_{2} s}} }}{\partial r} + \frac{{\partial^{2} C_{{CO_{2} ,s}} }}{{\partial z^{2} }}} \right] = V_{z,s} \frac{{\partial C_{{CO_{2} ,s}} }}{\partial z}$$

Velocity field is computed using^27^:

6 $$\rho \left( {V_{z - shell} \cdot \nabla } \right)V_{z - shell} = - \nabla \cdot \left[ { - pI + \mu \left( {\nabla V_{z - shell} + \left( {\nabla V_{z - shell} } \right)^{T} } \right)} \right]$$

where *p* is the pressure in the shell compartment of contactor. The density (*ρ*) and viscosity (*µ*) of the fluid is assumed to be constant. The symbol ${ }V_{z - shell} { }$ denotes the velocity in the axial coordinate. Also, the following equation is used for non-compressible fluid^27^:

7 $$\nabla \cdot V_{z - shell} = 0$$

### Membrane’s equations

The main equation for transport of CO_2_ through the fiber pores considering gas filled pores can be expressed as follows^27,28^:

8 $$D_{{CO_{2} ,mem}} \left[ {\frac{{\partial^{2} C_{{CO_{2} ,mem}} }}{{\partial r^{2} }} + \frac{1}{r}\frac{{\partial C_{{CO_{2} ,mem}} }}{\partial r} + \frac{{\partial^{2} C_{{CO_{2} ,mem}} }}{{\partial z^{2} }}} \right] = 0$$

Here, the fibers are considered to be filled with the gas phase due to the hydrophobic polymer used as the membrane and controlling the operational conditions^6^.

Diffusivity of CO_2_ in the membrane pores is obtained as^6,28^:

9 $$D_{{CO_{2} ,mem}} = \frac{{\varepsilon D_{{CO_{2} ,shell}} }}{\tau }$$

where tortuosity factor ($\uptau$) is calculated using the fiber porosity (ε)^29^:

10 $$\tau = \frac{{\left( {2 - \varepsilon } \right)^{2} }}{\varepsilon }$$

### Tube side's equations

Dispersion of CNT NPs will enhance the mass transfer rate in the system due to the synergistic effects. To consider Brownian and Grazing effects into developed mathematical model and simulation, several possible mechanisms were proposed in the literature for gas absorption in NFs. The diffusion coefficient for the MDEA-based nanofluid can be expressed as^30,31^:

11 $$D_{n,f} = D_{b,f} \left( {1 + m_{1} Re^{{m_{2} }} Sc^{{m_{3} }} \varphi^{{m_{4} }} } \right)$$

The modified diffusion coefficient is written as follows^30,31^ :

12 $$D_{n,f} = D_{b,f} \left( {1 + 640Re^{1.7} Sc^{1/3} \varphi } \right)$$

The symbol $\varphi$ denotes NP volume fraction in the liquid solvent. *Sc* (Schmidt) and *Re* (Reynolds) dimensionless numbers can be determined as follows^32^:

13 $$Re = \sqrt {\frac{{18KT\rho^{2} }}{{\pi d_{p} \rho_{p} \mu }}}$$

14 $$Sc = \frac{\mu }{\rho D}$$

where *K*, *d*_*p*_, and *D*_*b,f*_ are Boltzmann constant, NP diameter (8 nm), and carbon dioxide diffusion coefficient (1.45 × 10^–9^ m^2^/s), respectively.

To involve the Grazing effect in the developed model and its effect on CO_2_ mass transfer in the membrane contactor, the MDEA-based solvent phase was considered as two distinct solid and liquid and phases. Therefore, three mass transfer equations were derived and used in the developed model. Continuity equation of CO_2_ in the solid phase is derived as^22,33^:

15 $$\varphi \rho_{p} V_{z} \frac{\partial q}{{\partial z}} = k_{p} \alpha_{p} \left( {C_{{CO_{2} , tube}} - C_{s} } \right)$$

where *C*_*S*_ denotes CO_2_ concentration at the solid–liquid interface and *q* refers to the amount of CO_2_ adsorbed by the CNT NPs, which is determined with Langmuir isotherm^33^:

16 $$q = q_{m} \frac{{k_{d} C_{s} }}{{1 + k_{d} C_{s} }}$$

where q_m_ (29.45 mol/kg) refers to the maximum adsorption by CNT NPs, *k*_*d*_ (0.00049 m^3^/mol) is Langmuir constant. In Eq. 15, the symbol $\alpha_{p}$ is the specific surface area of NP and *k*_*p*_ is the mass-transfer coefficient between solid CNT nanoparticles and MDEA solvent, which is estimated as^33^:

17 $$Sh = \frac{{k_{p} d_{p} }}{{D_{{CO_{2} , tube}} }} = 2$$

The effective density of NF is written as^33^:

18 $$\rho_{L}^{nf} = \varphi \rho_{s} + \left( {1 - \varphi } \right)\rho_{L}^{bf}$$

In addition, the mass transfer equation for CO_2_ in the MDEA-based nanofluid (steady-state) and MDEA solvent is expressed as follows^27,33^:

19 $$D_{{{\text{CO}}_{2} ,tube}} \left[ {\frac{{\partial^{2} C_{{{\text{CO}}_{2} ,tube}} }}{{\partial r^{2} }} + \frac{1}{r}\frac{{\partial C_{{{\text{CO}}_{2} ,tube}} }}{\partial r} + \frac{{\partial^{2} C_{{{\text{CO}}_{2} ,tube}} }}{{\partial z^{2} }}} \right] = V_{z,tube} \frac{{\partial C_{{{\text{CO}}_{2} ,tube}} }}{\partial z} - R_{{{\text{CO}}_{2} }} + \frac{{k_{p} \alpha_{p} }}{1 - \varphi }\left( {C_{{CO_{2} , tube}} - C_{s} } \right)$$

20 $$D_{MDEA,tube} \left[ {\frac{{\partial^{2} C_{MDEA,tube} }}{{\partial r^{2} }} + \frac{1}{r}\frac{{\partial C_{MDEA,tube} }}{\partial r} + \frac{{\partial^{2} C_{MDEA,tube} }}{{\partial z^{2} }}} \right] = V_{z,tube} \frac{{\partial C_{MDEA,tube} }}{\partial z} - R_{MDEA}$$

Velocity distribution in the tube is determined using^27^:

21 $${\text{V}}_{{{\text{z}},{\text{tube}}}} = 2{\overline{\text{V}}}\left[ {1 - \left( {\frac{{\text{r}}}{{{\text{r}}_{1} }}} \right)^{2} } \right]$$

where ${\overline{\text{V}}}$ is the average velocity. MDEA structure, as well as the reaction rate of CO_2_ with MDEA are listed in Table 4. The reaction constant and reaction rate units are (m^3^ mol^−1^ s^−1^) and (mol^1^ m^−3^ s^−1^).

**Table 4:** The reaction rate between CO_2_—MDEA^34^.

Liquid absorbent	Molecular structure	Reaction rate
MDEA: CH_3_N [C_2_H_4_OH]_2_		$r_{{CO_{2} - MDEA}} = - 8.74110^{12} {\text{ exp}}\left( { - 8625/{\text{T}}} \right){\text{C}}_{{{\text{CO}}_{2} }} {\text{C}}_{{{\text{MDEA}}}}$

Table 5 provides the boundary conditions of mass and momentum transfer equation for the tube, membrane, and shell subdomains of the contactor.

**Table 5:** The boundary conditions of governing equations.

Position	Shell side		Membrane	Tube
	Mass	Momentum	Mass	Mass
*z* = 0	Convective flux	Outlet: pressure, no viscous stress, *p* = 0	Insulated	${\text{C}}_{{{\text{CO}}_{{2}} }}$ = 0, C_M_ = C_0_, *q* = 0
*z* = L	${\text{C}}_{{{\text{CO}}_{{2}} }}$ = C_0_	inlet velocity, V = V_0,shell_	Insulated	Convective flux
*r* = *0*	–	–	–	Axial symmetry
*r* = *r*_1_	–	–	${\text{C}}_{{{\text{CO}}_{{2}} }}$ = C_,tube_/*m*	${\text{C}}_{{{\text{CO}}_{{2}} }}$ = C_membrane_ × *m*, insulated
*r* = *r*_2_	${\text{C}}_{{{\text{CO}}_{{2}} }}$ = C_membrane_	No slip, wall	${\text{C}}_{{{\text{CO}}_{{2}} }}$ = C_shell_	–
*r* = *r*_3_	Insulated	No slip, wall	–	–

### Numerical solution

For solving the governing equations of MDEA-based solvent and gas phase, a finite element method is employed via COMSOL Multiphysics 5.4. The adaptive meshing and error control were employed, and PARDISO solver as one of the effective solvers for simulating membrane systems was employed to minimize the calculations errors^35,36^. It was found that the numerical solution time for solving the governing mass and momentum equations was about 3 min.

## Results and discussion

### Model validation

**Figure 3:**
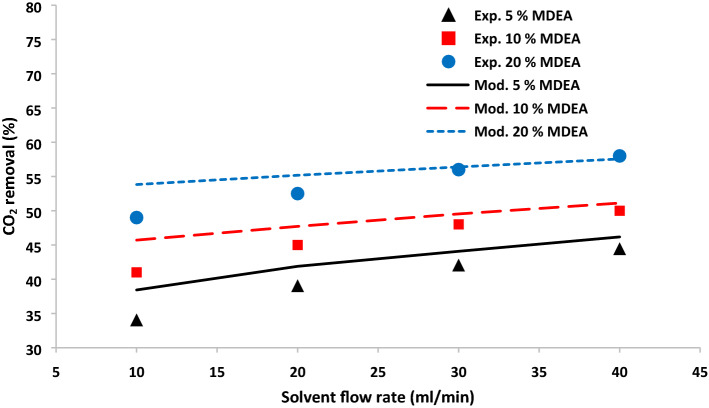
CO_2_ removal % as a function of solvent flow rate at different MDEA concentration without NPs at 10 ml/min gas flow rate.

**Figure 4:**
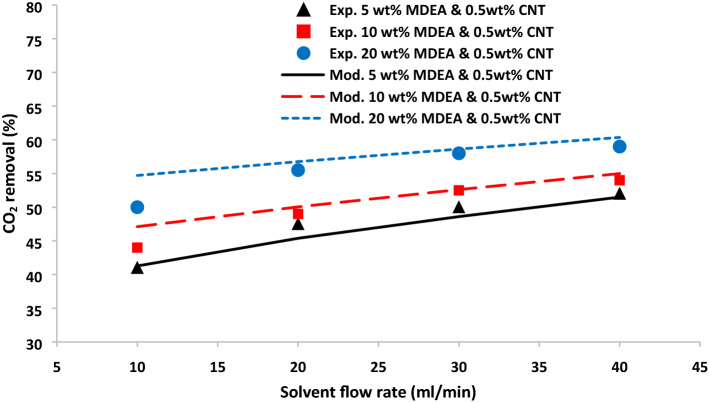
CO_2_ removal % as a function of solvent flow rate with 0.5 wt% CNT NPs at different MDEA concentration at 10 ml/min gas flow rate.

The mathematical model’s findings were verified by comparing with experimental data in terms of CO_2_ removal percentage. The CO_2_ absorption in the contactor module for different solvents containing MDEA reactant and MDEA-based nanofluid are presented in Figs. 3 and 4. There has been a great agreement between experimental data and modeling values for both membrane contactors with and without CNT nanoparticles. As observed, increasing solvent and MDEA-based nanofluid flow rate enhances the removal of carbon dioxide^37^. The liquid without NPs and MDEA-based nanofluid velocity can change the convection mass flux and consequently improves the overall mass transfer of carbon dioxide from the gas phase to the solvent phase. Figures 3 and 4 also indicated that MDEA-based nanofluid flow rate shows a considerable impact on the removal rate. In the system without NPs, increasing solvent flow rate from 10 to 40 ml/min led to the enhancement of carbon dioxide from 38.46 to 46.17%, 45.71 to 51.12%, and 53.82 to 57.56% for 5, 10, and 20% MDEA concentrations, respectively.

For the membrane contactor with 0.5 wt% CNT NPs, it is observed increase in CO_2_ removal from 41.28 to 51.49%, 47.11 to 55.00%, and 54.71 to 60.35% when MDEA-based nanofluid is increased from 10 to 40 ml/min for three different MDEA concentrations. Dispersion of 0.5 wt% CNT NPs into base fluid increases CO_2_ removal by 5.32, 3.88, 2.70% in the presence of 5, 10, and 20% MDEA when liquid flow rate is 40 ml/min. The effect of CNT NPs on CO_2_ removal is higher when there is lower MDEA concentration in the liquid phase.

### Gas flow rate effect on CO_2_ absorption

**Figure 5:**
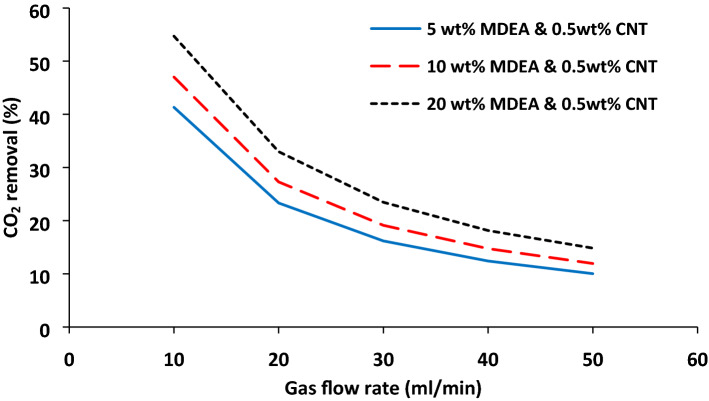
CO_2_ removal % as a function of gas mixture flow rate and MDEA concentration with 0.5 wt% CNT NPs at 10 ml/min MDEA-based nanofluid.

Based on the literature, one of the main advantages of the membrane technology is simple scale-up, since it is not difficult to find the surface area compared to conventional absorption processes. The enhancement of the gas flow rate can positively impact CO_2_ flux by reducing the mass transfer resistance. On the other hand, the residence time of the gas in the contactor is reduced with a rising gas velocity which results in the reduction of CO_2_ absorption efficiency. Figure 5 illustrates the influence of gas mixture flow rate on the carbon dioxide absorption at three different MDEA concentrations, including 5, 10, and 20 wt% As it was expected, increasing gas mixture flow rates from 10 to 50 ml/min reduces CO_2_ capture by 31%, 35%, and 40% in the presence of 0.5 wt.% CNT NPs and 5%, 10%, and 20% MDEA. Furthermore, increasing MDEA concentration increases CO_2_ removal. Given that the reaction kinetics of MDEA with carbon dioxide is of elementary type (Table 3), it depends directly on MDEA concentration.

**Figure 6:**
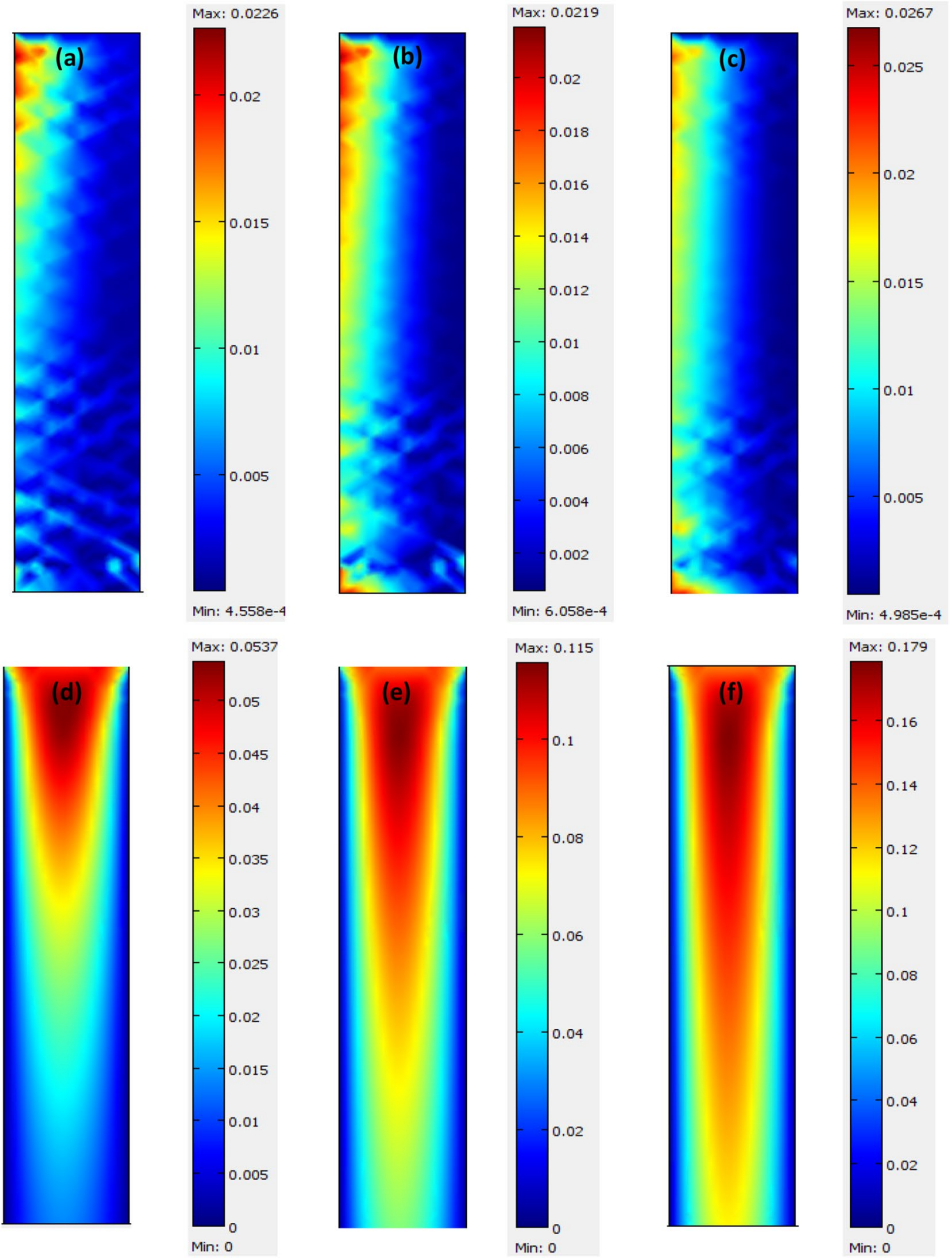
Effect of gas flow rate including 5 (**a**,**d**), 10 (**b**,**e**), and 15 (**c**,**f**) ml/min diffusive and convective CO_2_ flux in the shell subdomain of contactor; 0.5 wt% CNT NPs; solvent flowrate = 10 ml/min; (**a**–**c**) diffusive flux and (**d**–**f**) are convective flux.

CO_2_ diffusive and convective flux distribution in the gas on the shell subdomain of the contactor is illustrated for three different gas mixture flow rates, including 5, 10, 15 ml/min in Fig. 6. As it can be seen, the contribution of convective flux along the membrane contactor is significant in comparison with axial diffusive flux and the convective flux is maximum in the centre of the shell side where the gas velocity is maximum. This could be due to the fact that the velocity is predominant which can result in high convective flux^38^. Figure 6 illustrates that both diffusive and convective fluxes are reduced along the fiber length because of reducing the driving force in the *z*-direction. In addition, the convective flux is about 7 times higher than diffusive flux at higher gas flow rates.

### CO_2_ concentration distribution in the contactor

**Figure 7:**
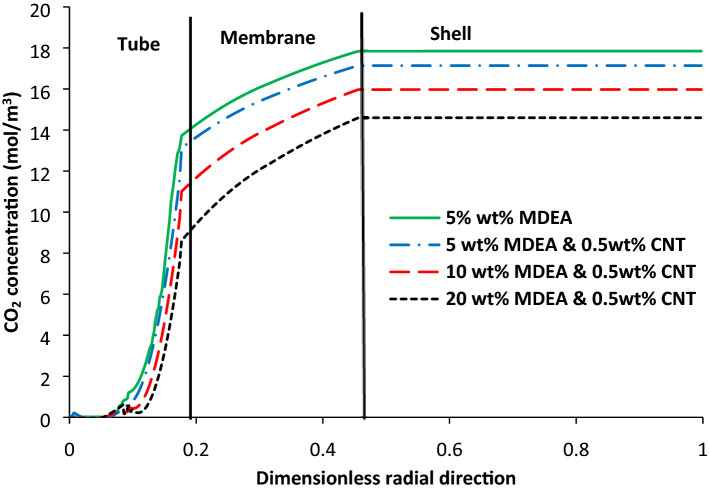
CO_2_ concentration profile in the radial direction of membrane contactor.

Concentration distribution of CO_2_ in 3 subdomains of the contactor at three different MDEA concentrations is indicated in Fig. 7. The concentration profile of CO_2_ is presented in r-direction. It is observed that concentration reduction of carbon dioxide in the microporous membrane subdomain, as well as the MDEA-based nanofluid, is significant, while there is not any significant change of the concentration profile in the shell side which gas stream flows. This phenomenon is due to the amount of diffusion coefficient in the gas phase, which is 10 and 10^4^ times higher than membrane and tube subdomains of membrane contactor, respectively. Moreover, it is indicated in Fig. 7 that CO_2_ concentration reduction in the membrane subdomain is higher when MDEA concentration is high. There is a sharp reduction in CO_2_ concentration near the membrane surface for all three different MDEA concentrations. As it can be seen, the change in CO_2_ concentration in the membrane subdomain is considerable, so it will be useful to investigate membrane specifications such as porosity and tortuosity on the CO_2_ absorption to enhance the separation efficiency.

### Effect of microporous membrane porosity and tortuosity

**Figure 8:**
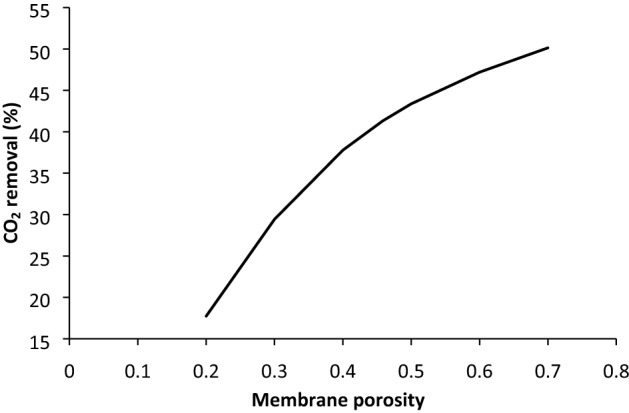
Effect of the membrane porosity parameter at constant tortuosity on the CO_2_ removal.

The effect of microporous membrane porosity on the CO_2_ removal using MDEA-based nanofluid is illustrated in Fig. 8. The fibre tortuosity was calculated using Eq. (10) for each amount of porosity. It was seen that the enhancement of the CO_2_ removal from gas stream can happen with increasing the membrane porosity. This is because of increasing diffusivity factor in the fibre pores with increasing porosity and consequently decrement of the mass transfer resistance for the transport of CO_2_ from gas to the solvent phase^39^. Furthermore, the slope of CO_2_ removal is sharper when the porosity is between 0.2 and 0.5, after that, the slope was decreased. It can be said that the used membrane (porosity = 0.4585) in this study is suitable for CO_2_ absorption using MDEA-based nanofluid. It should also be paid attention that with increasing porosity, the stability of membrane decreases, therefore, the optimum porosity should be selected based on operating condition. Moreover, the amount of tortuosity decreases with increasing the fibre porosity. It means that the removal percentage is decreased by enhancing the membrane tortuosity factor. Based on Eq. (9), the diffusion coefficient in the membrane subdomain is becoming less with increasing tortuosity factor and subsequently, the mass transfer resistance for CO_2_ diffusion through the microporous membrane would be increased^40,41^.

## Conclusion

A robust and reliable mechanistic model and simulation methodology was implemented to study the effects of CNT nanoparticles dispersion into MDEA-based solvent on the performance of the hollow fibre membrane contactor in terms of CO_2_ removal. In the current study, Brownian as well as Grazing mechanisms were taken into account as the main mechanisms of mass-transfer improvement in the separation system. There was a great agreement between the simulation results and experimental data reported in the literature. The CO_2_ removal increased from 41.28 to 51.49%, 47.11 to 55.00%, and 54.71 to 60.35% with the enhancement of MDEA concentration from 5 to 20% in the range of 10–40 ml/min MDEA-based nanofluid flow rate. The carbon dioxide absorption was increased by 14% with increasing porosity from 0.2 to 0.7, while it was decreased from 52.93% to 22.58% when the membrane tortuosity increased from 1 to 25. In addition, the main mass transfer resistance was in the tube subdomain where MDEA-based fluid flows.
